# Main characteristics and participation rate of European adolescents included in the HELENA study

**DOI:** 10.1186/0778-7367-70-14

**Published:** 2012-06-19

**Authors:** Laurent Béghin, Inge Huybrechts, German Vicente-Rodríguez, Stefaan De Henauw, Frédéric Gottrand, Marcela Gonzales-Gross, Jean Dallongeville, Michael Sjöström, Catherine Leclercq, Sabine Dietrich, Manuel Castillo, Maria Plada, Dénes Molnar, Mathilde Kersting, Chantal C Gilbert, Luis A Moreno

**Affiliations:** 1 Inserm U955, IFR 114/IMPRT, Faculty of Medicine, Université Lille Nord de France, F-59037, Lille, France; 2 CIC-PT- 9301-CH&U-Inserm of Lille, CHRU de Lille, F-59037, Lille, France; 3 Department of Public Health, Ghent University, B-9000, Ghent, Belgium; 4 GENUD (Growth, Exercise, NUtrition and Development) Research Group, Department of Physiotherapy and Nursing, School of Health Sciences, University of Zaragoza, Avd. Domingo Miral s/n, E-50009, Zaragoza, Spain; 5 Department of Health and Human Performance, Faculty of Physical Activity and Sport Sciences (INEF), Universidad Politécnica de Madrid, C/Martín Fierro, 7, E-28040, Madrid, Spain; 6 Laboratoire d’épidémiologie et de santé publique, Inserm U744, Institut Pasteur de Lille, Université Lille Nord de France, F-59024, Lille, France; 7 Department of Biosciences and Nutrition, Unit for Preventive Nutrition, Karolinska Institutet, Karolinska Institutet, S-10044, Huddinge, Sweden; 8 Istituto Nazionale di Ricerca per gli Alimenti de la Nutrizione, I-00178, Rome, Italy; 9 Medical University of Vienna, A-1040, Vienna, Austria; 10 Department of Medical Physiology School of Medicine, University of Granada, E-18071, Granada, Spain; 11 Faculty of Medicine, University of Crete, GR-710 03, Heraklion, Greece; 12 Department of Paediatrics, University of Pecs, H-7624, Pécs, Hungary; 13 Research Institute of Child Nutrition Dortmund, Rheinische Friedrich-Wilhelms-Universität Bonn, G-44225, Dortmund, Germany; 14 Department of Consumer & Sensory Sciences, Campden BRI, UK-GL1, Gloucestershire, United Kingdom

## Abstract

**Background:**

Participation rate and response rate are key issues in a cross sectional large-scale epidemiological study. The objective of this paper is to describe the study population and to evaluate participation and response rate as well as the key nutritional status variables in male and female adolescents involved in the HELENA study.

**Methods:**

A multi-stage random cluster sampling with a target sample of 3000 adolescents aged [12.5 to 17.5] years, stratified for geographical location and age, was carried out. Information for participants and non-participants (NP) was compared, and participation and response rates to specific questionnaires were discussed.

**Results:**

3,865 adolescents aged [12.5 to 17.5] years (1,845 females) participated in the HELENA study, of whom 1,076 (568 females) participated in the blood sampling. 3,528 (1,845 females) adolescents were finally kept for statistical analysis. Participation rates for the schools and classes differed importantly between countries. The participation rate of pupils within the participating classes also differed importantly between countries. Sex ratio, mean age and BMI were similar between NP and participating adolescents within each centre, and in the overall sample. For all the questionnaires included in the database, the response rate of questionnaires was high (more than 80% of questions were completed).

**Conclusion:**

From this study it could be concluded that participation rate differed importantly between countries, though no bias could be identified when comparing the key study variables between participants and non-participants. Response rate for questionnaires was very high. Future studies investigating lifestyle and health in adolescents can optimize their methods when considering the opportunities and barriers observed in the HELENA study.

## Background

Non-communicable diseases, including obesity, are the most common causes of morbidity and mortality in European adults, and they may originate in childhood and adolescence [1-3]. The complex interaction between genetics, growth and the environment that could be behind these diseases is poorly understood in adolescence. It has been hypothesised that a reduction in the risk factors for non-communicable diseases during growth may reduce morbidity and mortality impact later in life [4]. Major risk factors such as inadequate dietary habits [5-7] and physical inactivity [8-10] have been described. However, due to the complexity of the problem, the solution implies a collaborative and a multidisciplinary strategy to preserve a healthy physiological and psychological development during adolescence.

The first step towards addressing the problem is to acquire a reliable description of the current situation. The “Healthy Lifestyle in Europe by Nutrition in Adolescence” (HELENA) study ( http://www.helenastudy.com), is a European cross-sectional study (CSS) supported within the European Commission's 6^th^ Framework Program. The study aimed to obtain a complete picture of the nutritional status, body composition, lipids and metabolic profile, vitamin status, immune function related to nutritional status, physical activity and fitness levels, food choices and preferences, and genotype (analysing gene-nutrient and gene-environment interactions), by collecting data around 3000 adolescents in 10 European cities using the same methodology [11-13].

The aim of the present paper is to describe the study population and the key nutritional status variables in male and female adolescents involved in the HELENA study and compare basic variables (ie: gender and Body Mass Index) between participating to non-participating adolescents in HELENA study. These analyses were performed in order to check for possible selection bias.

## Methods

Prior to the initiation of the HELENA study, all methods were tested in a pilot study in order to test the sampling procedure, to improve blood transport logistics and analytics, and to optimise all questionnaires and exams [11]. In terms of study design, it was agreed that recruitment at a school setting would combine reliability (for comparisons) and feasibility (from a practical point of view). For practical reasons, and taking into account both educational and psychological considerations, complete school classes from the grades that best corresponded to the selected age group were selected.

### Study sample

The basis for the selection of the European cities was a practical one in the first instance, since it was not realistic to include a random sample of European adolescents given the timeline and budget available in the HELENA project.

#### Target population and sample size estimation

The aim of the HELENA study was to obtain a complete picture of the nutritional status, body composition, lipids and metabolic profile, vitamin status from a the target population: European adolescents [12.5–17.5] years old. Variance of BMI was chosen to calculate the sample size because this variable has the greatest dispersion in the study population with regards to the problem under consideration. The sample size was calculated with a confidence level of 95%, with ± 0.3 error in the parameter BMI. Error of 0.3 was chosen as a worst case scenario as precision level described by Cochran WG [14]. The number of adolescents to be studied was estimated at 3000 (300 in each centre). A subgroup of adolescents from the 10 cities was also randomly selected at class level (cf. *Infra*) to participate in blood sampling. We planed to analyse 74 blood parameters (excluding genetics) [15]. As 10 subjects per parameter are at least necessary to perform multivariate analysis, we considered than 740 subjects (74 × 10) were required for blood sampling and in order to prevent drop out, analysis/technical problems, we planned to include a bigger sample of 1000 adolescents.

#### Selection of the European cities

It was decided to study a city-based sample, including 10 European cities spread over different geographical areas across Europe striving for representativeness at the level of these cities. The cities were selected by convenience based on the location of the partners in the HELENA study consortium. Each city should have more than 100,000 inhabitants, with various cultural background and socioeconomic situation; presence of an active research group assuring sufficient expertise and resources to successfully perform studies in adolescents and an infrastructure allowing studies on adolescent health. Within the study, Stockholm (Sweden) represented Northern Europe; Athens and Heraklion (Greece), Roma (Italy) and Zaragoza (Spain) Southern Europe; Pécs (Hungary) Eastern Europe; and Ghent (Belgium) and Lille (France) Western Europe, and Dortmund (Germany), Vienna (Austria) Central Europe. In addition, an eleventh city, Birmingham UK, also participated in the HELENA CSS using the same selection and recruitment criteria. However, adolescents in the UK center only engaged in the study on food choices and preferences and therefore this sample has not been included in the descriptive statistics presented in this paper. A complete description of the HELENA study is available elsewhere [12,13]. Ethical issues, respect for good clinical practice and quality control procedures were addressed in a previous paper [16].

#### Selection of the study sample

A multi-stage random cluster sampling was carried out. Firstly, within the selected cities, schools were randomised taking into account several cluster stratifications: private/public school, location/area (zone or district), socio-economic level and age strata. The second step included randomisation of classes. In this random cluster sampling process, stratification was done at two levels, first at the level of cities and secondly at the level of school strata (as mentioned above). Within these strata the individuals are being selected in three stages. The first stage yielding schools as primary sampling units (PSU), and the second stage yielding classes as secondary sampling units (SSU). Individual pupils were then selected in the third stage as tertiary and final sampling units (TSU). Data concerning public/private school status, number of adolescents per class, and class level or grade, were provided by local school authorities. This procedure was carried out to ensure diversity of the sample in cultural and socioeconomic aspects.

The random selection of schools and classes was performed centrally (by the Ghent University) for almost all cities (except for Pecs and Athens, where schools were locally selected due to local administrative constraints). A list of 10 randomly selected schools was provided for each centre. At the same time, a replacement list including 20 substitute schools was also provided (replacement schools/classes were for the event of a school/class refusing to participate, and were from the same district and same class level/grade). Overall, 10 schools were involved in the survey in each city with the exception of Pecs where eight schools participated. The eligible school classes were then randomly selected from the list of school after stratification for grade. Up to three classes or class groups from two different grades were selected per school, with no more than 60 adolescents per school. In addition, 5 classes were randomly selected from 4–5 different schools for blood sampling. More details about the selection criteria for schools and classes were previously reported [12]:

The inclusion criteria in the study were:

Male and female subjects aged [12.5–17.5] years old.

Schooling in one of the participating classes.

Informed consent form signed by the parents and/or the legal guardian.

Subject was not participating simultaneously in another similar research.

Each participating centre was asked to include about 150 male and female adolescents per age stratum: [12.5-14], [14-15], [15-16], [16–17.5] years.

#### Recruitment strategy

Each research centre used the same procedure and documents (translated in local language; ie: information letter, consent form) for the adolescent recruitment process.

The first step of the recruitment strategy consisted in by a phone contact with the director/principal of the school. After this, a meeting with the director/principal and main/principal teachers of selected classes was organised in order to present aims, study procedure and require their agreement. The second step consisted in a meeting with adolescents from selected classes and their main/principal teacher. During this meeting (duration of 30 minutes to 1 hour), aims, study procedure and tests were explained. Information and consent forms were then distributed and the adolescents were asked to send their written/signed consent form (including signature of adolescent and both parents) within maximum two weeks after the meeting. If more than 80% of adolescents from a selected class accepted to participate to the study, class was included in the study. If the rate of acceptance was lower than 80% another class from the same school was randomly selected, then contacted.

#### Non participation rate

Adolescents were selected from participating school classes. In order to be as representative as possible of these European cities, schools and classes were randomly selected. This procedure of course implies non-participation (NP) which can introduce substantial error into the results, particularly when the participation rate is very low [17]. Moreover, it is difficult to provide information on the nature of the bias and its impact on the final results [18]. In any case, knowing NP rate is very important because low participation rates threaten the external validity of cross-sectional studies [19]. In this context, each research centre was asked to report the number of non-participating adolescents. Due to the two steps randomisation process, NP adolescents come from both non-participating and participating classes. For non-participating classes, it was impossible to collect any information about adolescents (due to ethical and practical points of view). However for NP from participating classes, at least gender, age, weight, and height were collected. These data were collected anonymously by the field workers using health records from school infirmary/nurse registries/databases, or via direct interview (phone contact) with the parents of NP adolescents using a specific anonymous abbreviated questionnaire, specially designed for this aim as part of the consent procedure.

This procedure was approved by Ethical committee from each country [16].

#### Response rate of questionnaires

Response rates were expressed as % of questions answered.

#### Selection of participants for statistical analysis

**Figure 1:**
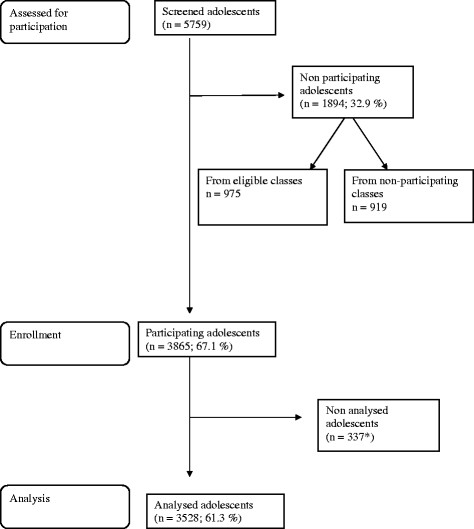
Flowchart of screening and participation rate in the HELENA study. * non analysed adolescents because of out of age range, missing of weight/height.

Exclusions for statistical analysis were carried out *a posteriori*, without the knowledge of the affected participants, to avoid feelings of discrimination in the excluded adolescents (since all adolescents from a class participated in the study, it was preferred not to exclude any subjects beforehand to avoid that some adolescents would feel discriminated). This procedure gave a final database of 3,528 adolescents eligible for statistical analysis (see Figure 1).

The exclusion criteria for statistical analysis were:

Male or female subjects aged < 12.5 or ≥ 17.5 years

Weight and/or height information missing

Having a chronic health problem was no an exclusion criteria.

### Methods and instruments used

#### Anthropometry

The anthropometric methods used within the HELENA study were described by Nagy *et al.*[20]. Briefly, body weight was measured in kg using a standard beam balance (Seca, precision 100 *g*, range 0–150 *kg*). Height was measured in cm using a precision stadiometer (Seca, precision 0.2 cm, range 70–200 cm). Body mass index (BMI in *kg/m²*) was calculated as weight (*kg*) divided by squared height (*m*^*2*^). Overweight was defined by means of the international BMI cut-off points for overweight and obesity proposed by The International Obesity Task Force (IOTF); the subjects were divided into overweight and obesity groups, according to the IOTF criteria published by Cole *et al.*[21]. Subjects with BMI higher than the equivalent 25 and 30 kg/m^2^ in adults were described as overweight and obese respectively.

#### Medical examination, blood sampling and questionnaires

Medical history, medication used, and all information as described below, were recorded in a specific case report form for each participant. Participants who were selected for the blood sampling were asked to abstain from eating and drinking after 8 p.m the day before the study. On the day of the study, a medical doctor either visited the school classes (in Athens, Dortmund, Ghent, Heraklion, Pecs, Roma, Stockholm, Vienna, Zaragoza), or participants went to a hospital ward (in Lille). Participants were asked for their medical history and recent acute diseases. A blood sampling questionnaire was used to assess fasting status, acute infections, allergies, smoking, vitamin and mineral supplements, and medication. Pubertal status was assessed by means of Tanner stages [22].

Blood pressure was measured with oscillometric devices of the same type and manufacturer (Omron M6) in all the study centres. Subjects sat quietly for 5 minutes, with their back supported, feet on the floor and right arm supported with the cubital fossa at the heart level. Two repeated recordings were made with 5 minutes in between and the lowest value of the 2 recordings of systolic blood pressure (SBP) and diastolic blood pressure (DBP) measurements was recorded.

Blood sampling generally took place between 8–10 a.m, after blood pressure measurement and body composition assessment by bio-impedancemetry [14]. Approximately 30 *mL* of blood was collected from an antecubital vein in serum and heparin monovettes® (Sarstedt AG & Co., Nümbrecht, Germany). Then, breakfast was offered to all participants. The blood sampling procedure within the HELENA study was described in detail by Gonzalez-Gross *et al.*[15]. Briefly, serum gel tubes were centrifuged at 3,500 rounds per minute (*rpm*) for 10 minutes within one hour after drawing blood. Within 24 hours serum was transported at room temperature to the central laboratory in Bonn. All samples were stored at −80°C until withdrawn for bunched analyses. Insulin (*μlU/mL*) was analyzed in plasma with IMMULITE® 2000 Advanced Immunoassay System (Siemens, Germany). Glucose (*mg/dL*), total cholesterol (TC; *mg/dL*), triglycerides (*mg/dL*), high density lipoprotein cholesterol (HDL; *mg/dL*), low density lipoprotein cholesterol (LDL; *mg/dL*), TC/HDL ratio, LDL/HDL ratio, glutamic oxaloacetic transaminase (*U/L*) and glutamic pyruvic transaminase (*U/L*) were analyzed in serum with the RxL clinical chemistry system (Dade Behring, Schwalbach, Germany).

All questionnaires were completed at school during specific time devoted to the study. Two or more fieldworkers were present in the classroom (*i*) to describe and explain questionnaires (*ii*) clarify any questions (*iii*) to assist if needed the adolescent to minimise non response rate.

#### Statistics

According to a Shapiro-Wilk test, all variables have normal distribution and are presented as means ± standard deviation. Prevalence for Tanner stages, overweight + obesity, and obesity, are displayed as percentages. Continuous variables were analysed by two-tailed *t*-test and categorical variables by the Chi-Square test.

A weighing factor for the whole sample, and a specific one for the subsample with blood assessments, was applied in order to adjust the final sample in the analyses to the theoretical distribution for age and gender. The SPSS 15.0 software for Windows (SPSS Inc, Chicago, Illinois) was used for the analyses and the significance level used was 5%.

## Results

### Participation rate and final database

Figure 1 presents a flow chart describing the recruitment process and expressing the participation rate. As whole classes were invited to take part, more subjects were included than was required from the sample size calculations, with a final sample of 3,865 adolescents included in the HELENA-CSS. Finally, 337 adolescents were not analysed because they were out of age range (n = 335), or have missing of weight/height (n = 2). Table 1 presents an overview of the participation rate of the different sampling units for the whole study and for each centre individually. The final database contains a total of 3,528 (1,845 females) adolescents for statistical analysis. The final sample kept for statistical analysis is presented in Table 2 and shows similar repartition of adolescents among gender and age strata.

**Table 1:** Number of approached/participating classes and adolescents in the HELENA study ^#^

**CENTERS**	**Athens**	**Dortmund**	**Ghent**	**Heraklion**	**Lille**	**Pecs**	**Roma**	**Stockholm**	**Vienna**	**Zaragoza**
Number of eligible schools in the city	82	55	43	22	40	12	290	25	347	83
Number of schools approached/participating	17/10	14/11	11/9	11/10	13/12	8/7	18/10	14/10	23/13	16/12
Number of classes approached/participating	14/14	23/23	20/19	22/20	19/18	24/14	24/22	25/23	35/19	26/23
Number of adolescents approached in all approached classes	458	603	429	429	538	720	470	645	870	597
Number of adolescents approached in all participating classes/adolescents participating	458/370	603/515	413/347	400/340	508/308	420/401	430/339	535/377	536/427	537/441
Number of adolescents eligible for statistical analysis	321 (70%)*	476 (79%)*	336 (78%)*	284 (66%)*	287 (53%)*	394 (55%)*	304 (65%)*	341 (53%)*	403 (63%)*	382 (64%)*
**BLOOD SAMPLE**										
number of classes approached/participating for blood sample	6/6	8/8	6/6	8/8	8/6	5/5	10/9	8/5	10/7	7/7
number of adolescents approached/participating for blood sample	161/137	155/136	145/125	142/122	177/108	150/141	186/112	150/128	198/125	136/125
Number of adolescents eligible for statistical analysis	104 (65%)*	122 (79%)*	113 (78%)*	104 (85%)*	86 (49%)*	136 (91%)*	101 (55%)*	99 (66%)*	112 (56%)*	112 (82%)*

^#^ Data collected from 2006 to 2007.

* Percentage calculated to reflect ratio of selected adolescents for statistical analysis to adolescents approached in all approached classes.

**Table 2:** Repartition of number of adolescents by gender and age strata in the HELENA study final sample ^§ #^

**Age strata*(Years)***	**Females**		**Males**	
	**n**	**%**	**n**	**%**
[12.5-14]	**610**	33.1	**530**	31.5
[14-15]	**466**	25.3	**409**	24.3
[15-16]	**440**	23.8	**417**	24.8
[16–17.5]	**329**	17.0	**327**	19.4

^§^ adolescents were included in ten cities: Athens in Greece, Dortmund in Germany, Ghent in Belgium, Heraklion in Greece, Lille in France, Pecs in Hungary, Roma in Italy, Stockholm in Sweden, Vienna in Austria and Zaragoza in Spain. ^#^ Data collected from 2006 to 2007.

The participation rate for the schools and classes differed importantly between countries (with Austria having the lowest participation rate: 57%, and France the highest: 92% for schools). The participation rate for pupils within the participating classes also differed importantly between countries (ranging from 61% for France to 95% for Hungary). Blood sampling was performed in one third of the adolescents recruited (selection of entire classes representing the desired age of the adolescents); in total, 1,076 adolescents (568 females) participated in the blood sampling. For most countries, the participation rate for the adolescents invited to participate in the blood sample subgroup was similar to the total participation rate in that country (except for Italy where the participation rate was 80% for the total sample versus 60% for the adolescents giving blood samples).

### Comparison of characteristics between participants and non-participants

Table 3 presents information related to gender, age, weight, and height collected in NP subjects from participating classes. It was not possible to collect this information in non-participating classes because in this case the class was excluded before any field work started and we were not allowed by school authorities to have any contact with these adolescents or their parents. In some occasions, information about NP subjects from participating classes was also lacking because teachers or school directors did not want to provide any data from these adolescents, to respect the NP’s privacy and their decision not to participate. In total we could collect complete information from 710 NP adolescents out of the 975 NP from the participating classes. Sex ratio, mean age and reported BMI were similar between NP and participating adolescents within each centre, and in the overall sample (Table 3).

**Table 3:** Characteristics of the 710 non-participating adolescents from participating classes compared to participating adolescents in HELENA study ^#^

**CITIES**	**Sex ratio (M/F)**		**Age*(years) (mean ± SD)***		**BMI (*kg/m***^***2***^) (***mean ± SD*)**	
	**Non-participants**	**Participating adolescents**	**Non-participants**	**Participating adolescents**	**Non-participants**	**Participating adolescents**
Athens	**NA**	51/49	**NA**	13.9 ± 1.2	**NA**	21.8 ± 3.8
Dortmund	**40/60**	43/57	**15.0 ± 1.0**	15 ± 1.4	**21.2 ± 5.2**	21.8 ± 4.2
Ghent	**49/51**	54/46	**15.0 ± 1.5**	15 ± 1.3	**20.9 ± 3.6**	20.3 ± 3.2
Heraklion	**NA**	54/46	**NA**	14 ± 1.3	**NA**	23.2 ± 4.5
Lille	**57/43**	58/42	**13.8 ± 1.4**	14.4 ± 1.2	**19.5 ± 6.2**	20.5 ± 3.7
Pecs	**NA**	50/50	**NA**	15 ± 1.2	**NA**	21.4 ± 3.6
Roma	**40/60**	60/40	**15.2 ± 1.6**	15.5 ± 1.7	**21.8 ± 4**	22.2 ± 4.0
Stockholm	**47/53**	61/39	**14.1 ± 1.2**	15 ± 1.4	**20.9 ± 3.2**	20.5 ± 3.2
Vienna	**44/66**	51/49	**15.1 ± 1.4**	15.3 ± 1.5	**22.2 ± 5.1**	21.7 ± 3.7
Zaragoza	**43/57**	51/49	**14.6 ± 1.2**	14.6 ± 1.3	**20.5 ± 1.9**	21.6 ± 3.3
**TOTAL**	**320/390**	53.3/46.7^NS^	16.6 ± 1.2**	14.8 ± 1.2^NS^	21.1 ± 4.2**	21.5 ± 3.9**^NS^

^#^ Data collected from 2006 to 2007.

NA = Not Available; NS : Not significant for *t*-test; ** for available data.

### Response rate for questionnaires

The HELENA study contains 10 different questionnaires addressing a range of topics including perceived health, socio-economic status, and adolescents’ knowledge and attitudes towards nutrition and physical activity. Participating adolescents were invited to answer 474 questions in total. Our challenge was to obtain maximum cooperation from the adolescents in terms of understanding the questions, in order to minimize the risk of non-response and errors [23]. Table 4 presents the number of questions in each questionnaire, percentage of questionnaires present in the database (whether the questionnaire was totally completed or not by the adolescents), and percentage of answered questions within questionnaires. Percentage of questionnaires present in the database differed according to the type of questionnaire and ranged from 83.6% for the larger Food Choices and Preferences questionnaire (FCPq), to 97.2% for both the Nutrition knowledge test (NKT) and the General questionnaire-Socio Economic Status (GQ-SES). More than 83% of the adolescents agreed to fill in the questionnaires. The relatively high rate of missing Parental Questionnaires (PQ) (about 20%) is easily explained by the fact that this questionnaire was addressed to the parents who had to send it back to the field worker at the time of the study. For all questionnaires included in the database, the response rate was high, more than 80%. In general, we can consider that once an adolescent decided to respond to a given questionnaire, he/she was generally quite thorough and responded to the majority of the questions.

**Table 4:** Completion and response rates for the HELENA study questionnaires ^§ #^

**Questionnaire**	**n of questions**	**% of questionnaires in the database **^**1**^	**% of questions answered in each questionnaire **^**2**^
GQ-SES	27	97.2	95.5
NKT	46	97.2	98.4
EWI	60	96.4	99.6
FCP	196	83.6	98.1
HE	41	96.6	96.5
PA	40	92.5	96.0
PAQ	22	87.5	99.9
SQ	22	96.1	98.8
PQ	5	80.9	98.4

^§^ adolescents were included in ten cities: Athens in Greece, Dortmund in Germany, Gent in Belgium, Heraklion in Greece, Lille in France, Pecs in Hungaria, Roma in Italy, Stockholm in Sweden, Vienna in Austria and Zaragoza in Spain. ^#^ Data collected from 2006 to 2007.

^1^ Ratio of number of questionnaires available in the database to the expected number of questionnaires (according to the number of adolescents included).

^2^ Ratio of answered questions available in the database to the expected number of questions.

GQ-SES: General questionnaire - Socio economic status questionnaire NKT: Nutrition knowledge test EWI: Eating behavior and weight problems inventory FCP: Food choices and preferences questionnaire HE : Healthy eating determinants PA: Physical activity determinants PAQ: Physical activity questionnaire SQ: Sedentary questionnaire PQ: Parental questionnaire.

### Migrant status

The socio-economic questionnaire addressed some questions about origins of adolescents included in HELENA. Migrant adolescents were defined as adolescents born outside the country where they lived. A similar definition was applied for their parents. The proportion (according to total population in HELENA sample) of migrant adolescents (Table 5) varied widely according to each city from 0.6 to 9% for the adolescents, and from 2.4 to 22.8% for their parents. Main countries of origin for migrant subjects and their parents were: Turkey, Lebanon, Morocco, Algeria, Serbia, Poland, Ecuador, Slovenia, Romania, Albania, and Russia.

**Table 5:** Proportion of migrants by study centre in HELENA study ^#^

**CITIES**	**% of migrant adolescents**^**Ω**^	**% of migrant parents**^**Ω**^
Athens	0.6	22.8
Dortmund	5.1	15.5
Ghent	3.6	10.1
Heraklion	1.7	2.4
Lille	3.4	11.5
Pécs	4.0	4.7
Roma	6.3	7.6
Stockholm	9.0	11.3
Vienna	2.0	14.5
Zaragoza	4.8	7.9
**TOTAL***Mean ± SD*	**4.0 ± 2.4**	**10.8 ± 5.8**

^#^ Data collected from 2006 to 2007.

^**Ω**^ Migrant adolescents were defined as adolescents born outside the country where they lived. A similar definition was applied for their parents.

### Description of main anthropometric and biochemical variables

Descriptive data for the study sample is presented in Table 6. Compared to males, females were 8 cm shorter and 7 kg lighter, and showed a lower systolic blood pressure (all p < 0.05, Table 6). Females were on average in a more advanced stage of sexual maturation and their prevalence of overweight + obesity, and only obesity, were lower than in males (6.9 and 3.5 points of % respectively; p < 0.05, Table 6). Serum concentrations of the main biochemical variables for the study sample are displayed in Table 7. Compared to males, females exhibited lower glucose, and both glutamic oxaloacetic and glutamic pyruvic transaminases concentrations (all p < 0.05, Table 7), but higher total cholesterol, triglycerides, and both high and low density lipoprotein cholesterol concentrations (all p < 0.05, Table 7).

**Table 6:** Descriptive data for female and male adolescents in HELENA study ^§ #^

	**Female**				**Male**				**Total**			
	**n**	**mean**	**±**	**SD**	**n**	**mean**	**±**	**SD**	**n**	**mean**	**±**	**SD**
Age (*yr*)	1829	15.0	±	1.2	1659	15.0	±	1.2	3488	15.0	±	1.2
Height (*cm*)	1845	162.3^ψ^	±	6.8	1683	170.8	±	9.4	3528	166.4	±	9.1
Weight (*kg*)	1845	56.4^ψ^	±	10.1	1683	63.4	±	14.3	3528	59.5	±	12.7
BMI (*kg/m*^*2*^)	1845	21.4	±	3.5	1683	21.6	±	3.9	3528	21.5	±	3.7
Systolic blood pressure (*mm Hg*)	1813	117.3^ψ^	±	12.8	1631	127.1	±	15.6	3444	122.0	±	15.0
Diastolic blood pressure (*mm Hg*)	1813	69.2	±	10.3	1631	68.7	±	10.5	3444	69.0	±	10.4
Tanner stage (1/2/3/4/5) (*n*)	0/46^ψ^/308/745^ψ^/542^ψ^				10/100/310/604/466/							
%	0/2.8^ψ^/18.8/45.4^ψ^/33.0^ψ^				7.0/6.70/20.8/40.5/31.3							
Overweight + obesity ^Φ^(*%*)	19.5*				26.4							
Obesity (%)	3.8*				7.3							

^§^ adolescents were included in ten cities: Athens in Greece, Dortmund in Germany, Ghent in Belgium, Heraklion in Greece, Lille in France, Pecs in Hungary, Rome in Italy, Stockholm in Sweden, Vienna in Austria and Zaragoza in Spain. ^#^ Data collected from 2006 to 2007.

^Φ^Overweight and obesity were defined uses IOTF cut off values [21].

^ψ^P < 0.05 for comparison between females and males (*t*-test and *χ*2).

**Table 7:** Serum concentrations for the main biochemical characteristics in female and male adolescents in HELENA study ^§ #^

	**Female**				**Male**				**Total**			
	**n**	**mean**	**±**	**SD**	**n**	**mean**	**±**	**SD**	**n**	**mean**	**±**	**SD**
Glucose (*mg/dL*)	568	89.2^ψ^	±	6.8	508	92.7	±	7.3	1076	90.9	±	7.2
Insulin (*μlU/mL*)	555	10.2	±	6.4	499	10.0	±	8.6	1054	10.1	±	7.5
Total cholesterol (TC) (*mg/dL*)	568	167.1^ψ^	±	27.9	508	153.4	±	25.6	1076	160.6	±	27.7
Triglycerides *(mg/dL*)	568	73.5^ψ^	±	37.6	508	65.0	±	31.7	1076	69.5	±	35.2
High density lipoprotein cholesterol (*mg/dL*)	568	56.9^ψ^	±	10.9	508	52.7	±	9.9	1076	55.0	±	10.6
Low density lipoprotein cholesterol (*mg/dL*)	568	98.0^ψ^	±	25.1	508	90.4	±	23.8	1076	94.4	±	24.8
TC/HDL ratio	568	3.0	±	0.6	508	2.9	±	0.6	1076	3.0	±	0.6
LDL/HDL ratio	568	1.7	±	0.5	508	1.7	±	0.6	1076	1.7	±	0.6
Glutamic oxaloacetic transaminase (*U/L*)	568	20.0^ψ^	±	7.2	508	24.1	±	6.8	1076	21.9	±	7.3
Glutamic pyruvic transaminase (*U/L*)	568	19.7^ψ^	±	8.2	508	23.4	±	9.2	1076	21.5	±	8.9

^§^ adolescents were included in ten cities: Athens in Greece, Dortmund in Germany, Ghent in Belgium, Heraklion in Greece, Lille in France, Pecs in Hungary, Rome in Italy, Stockholm in Sweden, Vienna in Austria and Zaragoza in Spain. ^#^ Data collected from 2006 to 2007.

^ψ^P < 0.05 for comparison between females and males (*t*-test).

## Discussion

### Sample

In our study the participation rate for the schools and classes differed importantly between countries as was also observed by Klepp *et al.*[24]. We observed a 67% mean participation rate for adolescents in our study, which can be considered acceptable for such a demanding epidemiological study. Indeed, the participation rate in our study was close to those from other comparable epidemiological studies such as the MONICA study: 77.2% [25] the study of Hill *et al.*: 75.4% [26] and 74.7% [27] obtained in the ProChidren study. Participation rate varied widely among cities, this was probably due to (*i*) time constraints about study procedure perceived differently among cities (*ii*) different national/local school organisation and (*iii*) knowledge gap between countries regarding parental perceptions and attitudes toward their children’s participation to a research project [28,29]. Moreover, the sampling procedure used in the HELENA study contributed to an increase in the non-participation rate. This was because classes that had less than 70% of adolescents who wanted to participate in the study were entirely excluded, thereby excluding individuals in these classes who were willing to participate. Since we were able to collect data from the non-participating adolescents from participating classes, we could check whether these subjects had roughly the same characteristics as those who did participate, and therefore whether there were any sampling errors or bias in the selection of HELENA study subjects. Our data show no signs for a significant bias in the participating sample for the key variables in our study (Table 3).

### Completion rate and response rate of the questionnaires

The percentage of missing questionnaires was low in our study and the majority of the questionnaires included in the database had a high rate of completion since most of the questions were answered as was also observed by Klepp *et al.*[24]. This is a critical point for our study where a large number of self-administered questionnaires were applied, with a risk of weariness and discouragement for the adolescents. In any case, non-responses should not affect data quality when they occur randomly [18,30]. Our study is one of the first to report reliability of adolescents for filling out questionnaires. We clearly demonstrated that a good completeness can be obtained in this age group and therefore that results obtained are representative of the total sample of adolescents who participated in the HELENA study.

### Clinical and biological characteristics

Our sample was balanced in terms of repartition and age strata, sex and BMI. To compare BMI between participating and non-participating adolescents, we used weight and height collected from health records from school infirmary registries or self reported data. As weight and height from participating adolescents were measured, this comparison could be biased (direct *vs* indirect measurement).

SBP and DBP were measured according to methods described in a previous publication [31] Mean SBP and DBP were relatively high compared to data obtained in adolescents studied by Krzyzaniak A *et al.*[32]. Using cut off values from National High Blood Pressure Education Program Working Group on High Blood Pressure in Children and Adolescents [33] for SBP, we found a unexpectedly high percentage of hypertensive adolescents (*i.e.* 28.15%; data not shown). As all adolescents included in the HELENA study were presumed healthy, the high percentage of hypertensive adolescents was probably due to methodological bias (white coat effect, stress, few numbers of measures, etc.…). This issue should be clarified by undertaking further analysis. As expected, pubertal status was more advanced in girls than boys, which is normal for these age ranges. Ratio of overweight and obese adolescents was within habitual range according to Lobstein T *et al.*[34]. Serum concentrations for the main biochemical characteristics were also in the normal range [33,35,36]. The most striking results in this regard were the higher values in girls for all of the studied lipid-related variables. These results are in accordance with results observed in Turkish children and adolescents aged 7–18 years [37]. However, Spanish girls had only high total cholesterol and high density lipoprotein cholesterol compared to their male counterparts [38]. Differences between studies may be due to genetic variation and/or different eating behaviors between countries; methodological aspects such as age categorizations and age ranges may contribute to discrepancies. The HELENA study, because of its multi-country design, can minimize these effects.

## Conclusion

The HELENA study offers the opportunity to study the interaction of nutrition and health in a European adolescent population. This study provides, for the first time, a complete picture of their nutrition and health status using high quality data capturing and reporting processes. We clearly demonstrated that a good completeness and a high quality of data can be obtained in this age group. In our study, non-participating adolescents appeared not differ significantly from the participants in the key variables and no sign of selection bias was identified.

Several obstacles, mainly due to the multinational design of the study, were encountered: (*i*) difference in regulatory requirement among countries [16]; (*ii*) multilanguage’ questionnaires leading to a long process of translation/crosstranslation [31], (*iii*) national food composition and habits requiring to adapt software and use national food libraries [39], (*iv*) harmonisation and accuracy of measurement procedures/tools that needs training and harmonization [20,40], (*v*) logistics of blood samples through Europe that require precise treaçability [15].

In addition, the practicalities of different aspects included in the study were tested at a pan-European level and were described and discussed in this paper so that future studies dealing with obesity in adolescents and other related factors, can optimize their methods when considering the opportunities and obstacles observed in the HELENA study.

## Competing interests

Frédéric Gottrand has received consulting fees from Numico Clinical Nutrition, lecture fees from SMS and grant support from Danone Research. The remaining authors declare that they have no competing interests.

## Authors’ contributions

LAM coordinated the HELENA project on international level. LAM, SD, FG, MGG, JD, MS, CL, MC, DM, MK and CCG were involved in the design of the HELENA project and locally coordinated the HELENA project. LB, GVR, SB, and MP organized the fieldwork and performed the data collection locally. IH was responsible for the database management. LB was responsible for regulatory issues. LB, GVR and IH drafted the article. All authors read and approved the final manuscript.
